# Genotype-specific antioxidant, reactive-carbonyl, and polyamine responses underlying heat tolerance in six winter wheat cultivars

**DOI:** 10.3389/fpls.2026.1973386

**Published:** 2026-10-26

**Authors:** Altafur Rahman, Yawar Habib, Andrea Jara Quispe, Jhohan Piero Elguera, Hedvig Krecsák, Imran Khan, Magda Pál, Krisztina Balla, Tsanko Gechev, Imre Majláth

**Affiliations:** 1 Center of Plant Systems Biology and Biotechnology, Plovdiv, Bulgaria; 2 Center for Bio and Medical Technologies, Skolkovo Institute of Science and Technology, Moscow, Russia; 3 Hungarian University of Agriculture and Life Sciences, Budapest, Hungary; 4 HUN-REN ATK, Agricultural Institute, Martonvásár, Hungary; 5 Department of Microbiology, Eötvös Loránd University, Budapest, Hungary; 6 Department of Molecular Biology, University of Plovdiv, Plovdiv, Bulgaria

**Keywords:** antioxidant enzymes, ascorbate–glutathione cycle, heat stress, polyamines, reactive carbonyl species, stress-tolerance index, *Triticum aestivum*

## Abstract

Heat stress is a major constraint on wheat (*Triticum aestivum* L.) production, intensifying with climate change. Tolerance differs widely among cultivars, yet how the antioxidant, reactive-carbonyl, and polyamine systems are jointly reconfigured as temperature increases is unclear. We profiled six winter wheat cultivars at 20 °C, 35 °C, and 40°C. We measured shoot biomass, pigments, and gas exchange; assayed the activities of the ascorbate–glutathione and reactive-carbonyl detoxification enzymes and phenylalanine ammonia-lyase; and quantified major polyamine pools. We then integrated these data using principal component analysis (PCA) and a stress-tolerance index (STI). Biomass, chlorophyll, and photosynthesis declined with temperature, whereas carotenoids and defensive enzymes were induced at 35°C and anthocyanins at 40 °C. Cultivars separated at 40 °C: tolerant genotypes maintained their defences, sensitive ones did not. Two signatures were most diagnostic: glyoxalase I and alkenal/alkenone oxidoreductase remained above control in every cultivar except Mv Tarsoly, the only genotype in which both carbonyl-detoxification arms declined below baseline. Cadaverine, a rarely reported diamine, also peaked in Mv Tarsoly, which ranked lowest in both PCA and STI. Spermine and spermidine decreased by up to 77% and 57%, against several-fold increases in putrescine, cadaverine, and 1,3-diaminopropane, with putrescine peaking in Mv Ikva. Polyamine remodelling thus acted as a marker of oxidative stress and, we propose, may also mediate it. PCA and STI supported this pattern, ranking Mv Lucilla, Mv Kolompos, and Mv Dandár above Mv Pirkadat, Mv Ikva, and Mv Tarsoly; the first three are candidate germplasm for climate-tolerant wheat breeding, pending field validation.

## 1 Introduction

Wheat (*Triticum aestivum* L.) is one of the most important cereals in the world and a cornerstone of global food security, but its productivity is increasingly threatened by temperature stress (Zheng et al., 2025; Lamba et al., 2023). Increasingly frequent heatwaves associated with ongoing climate change, especially when they coincide with sensitive developmental stages, are reducing grain yields and quality and are destabilising wheat production in the main growing regions (Lamba et al., 2023; Zheng et al., 2025; Fathi et al., 2025). As options to mitigate increasing field temperatures are limited and heat episodes become more erratic, the development and selection of heat-tolerant cultivars is now dependent on a mechanistic understanding of how the different genotypes perceive and respond to thermal stress at physiological and biochemical levels (Zheng et al., 2025).

At the cellular level, supraoptimal temperatures impair photosynthesis, destabilise membranes and proteins, and induce overproduction of reactive oxygen species (ROS), which oxidise lipids, proteins, and pigments, disrupt the thylakoid and plasma membranes, and inactivate the photosystems and Rubisco (Iqbal et al., 2021; Zheng et al., 2025; Fathi et al., 2025). High temperature also reduces chlorophyll content and the chlorophyll *a*/*b* ratio, a marker of light-harvesting antenna size, although tolerant genotypes retain higher pigment levels and greater gas-exchange capacity (Sharma et al., 2015). ROS-driven lipid peroxidation further generates cytotoxic reactive carbonyl species (RCS) such as methylglyoxal and malondialdehyde, which damage proteins and nucleic acids and are removed by detoxification systems comprising the glyoxalase (GLY) system, including GLY I (EC 4.4.1.5), the nicotinamide adenine dinucleotide phosphate (NADPH)-dependent aldo-keto reductases [AKRs, including aldose reductase (EC 1.1.1.21)] and alkenal/alkenone oxidoreductases (AOR/AERs; EC 1.3.1.74.). These systems remain far less studied than the classical antioxidants in cereal heat tolerance (Hasanuzzaman et al., 2017; Soccio et al., 2022; Rathore et al., 2024). The enzymatic antioxidant defence itself centres on the ascorbate–glutathione (AsA–GSH) cycle [ascorbate peroxidase (APX; EC 1.11.1.11), dehydroascorbate reductase (DHAR; EC 1.8.5.1), and glutathione reductase (GR; EC 1.8.1.7)] and glutathione *S*-transferases (GST; EC 2.5.1.18). These enzymes are upregulated in heat-stressed wheat shoots to a genotype-dependent degree (Goyal and Asthir, 2010; Iqbal et al., 2021; Kumar et al., 2023). This enzymatic defence is complemented by nonenzymatic carotenoids, phenylpropanoids, and anthocyanins, with anthocyanin biosynthesis regulated by phenylalanine ammonia-lyase (PAL; EC 4.3.1.24) (Mohi-Ud-Din et al., 2021; Rahim et al., 2023; Karamat et al., 2024).

Polyamines constitute a further, distinctive layer of protection that has been a particular focus of cereal stress-physiology research at Martonvásár (Pál et al., 2015, 2022). The higher polyamines spermidine (Spd) and spermine (Spm) and the diamine putrescine (Put) stabilise membranes and nucleic acids, scavenge ROS, and modulate signalling; their pool is maintained at a set point by biosynthesis and catabolism (Pál et al., 2015; Jing et al., 2020). Under heat stress, this balance shifts towards catabolism: flavin-dependent polyamine oxidases (PAO) either back-convert the higher polyamines or degrade them terminally, with terminal degradation yielding 1,3-diaminopropane (DAP), while copper-containing diamine oxidases (DAO) oxidise putrescine; these reactions release hydrogen peroxide, which acts as a signalling molecule (Gholizadeh and Mirzaghaderi, 2020; Napieraj et al., 2023; Samanta et al., 2023; Pál et al., 2018; Rahman et al., 2024). Free putrescine and spermidine accordingly decline under heat while genes involved in catabolism are induced (Upadhyay et al., 2020); in wheat, moderate heat lowered the higher polyamines while raising DAP in parallel with a polyamine-oxidase gene (Janda et al., 2019). This shift towards the diamines is used as a sensitive indicator of stress severity (Jing et al., 2020; Pál et al., 2018). In wheat, however, it is not known how the complete polyamine pool changes between cultivars and between moderate and severe heat.

Heat tolerance is a quantitative, genotype-dependent trait. Tolerant genotypes sustain photosynthesis and have a more effective defence system, which limits their oxidative and carbonyl load, whereas sensitive genotypes accumulate more damage (Mohi-Ud-Din et al., 2021; Goyal and Asthir, 2010). No single trait captures this complexity; therefore, contrasting cultivars are increasingly screened using multivariate analyses and stress-tolerance indices (Lamba et al., 2023). The Martonvásár (‘Mv’) winter wheat cultivars are widely grown in Central Europe and have been characterised for drought and CO_2_ responses (Farkas et al., 2021; Farkas and Varga, 2025), for yield-level heat responses (Balla et al., 2019, 2021) and for polyamine and antioxidant responses to abiotic stress (Pál et al., 2013; Janda et al., 2019). However, an integrated profile across these biological systems under graded heat is still lacking for these genotypes.

In this study, six winter wheat cultivars (Mv Tarsoly, Mv Kolompos, Mv Lucilla, Mv Pirkadat, Mv Ikva, and Mv Dandár) were tested at 20 °C, 35 °C, and 40 °C. Shoot biomass, photosynthetic pigments, leaf gas exchange, AsA–GSH enzymes (APX, DHAR, GR), reactive-carbonyl detoxification enzymes (GST, AKR, AOR/AER, GLY I), PAL, and five major polyamines were examined using PCA and a trait-wise STI analysis to resolve the dose-dependent response, rank cultivars for heat tolerance, and characterise how the polyamine pool is remodelled across genotypes and temperatures. The specific objectives were therefore fourfold: to establish how the antioxidant, reactive-carbonyl, and polyamine systems respond to a graded increase in temperature; to determine which of these responses differ between cultivars and therefore distinguish tolerant from sensitive genotypes; to identify a small set of biochemical markers by which heat tolerance in winter wheat could be screened; and to provide an experimental basis for predicting how potential heatwaves in late autumn and early spring would affect the stress response of Central European winter wheat.

## 2 Materials and methods

### 2.1 Plant growth

Hungarian winter wheat cultivars, Mv Dandár, Mv Lucilla, Mv Pirkadat, Mv Tarsoly, Mv Ikva, and Mv Kolompos (*Triticum aestivum* L.), were used for the experiments. Following the germination phase, the seedlings were transferred to a mixture of chernozem soil and sand (3:1 v/v). A total of seven plastic pots were used for each temperature, with 12 seeds of one cultivar sown into each. The plant growth and stress treatments were then carried out in Faithful FPG-450 growth chambers (Huanghua Faithful Instrument Co., Huanghua, China) with cultivars randomised within each chamber. The experiment comprised two consecutive phases. During the first phase, all seedlings were grown for three weeks under optimum conditions [control, 20/16 °C L/D (light/dark), PPFD = 250 µmol m^−2^ s^−1^, 16/8 h L/D cycle and standard irrigation at 65/75% RH L/D], until tiller formation. During the second phase, which lasted 2 weeks, the control plants remained under these conditions, while the other plants were exposed to moderate heat (35/20 °C L/D) or severe heat (40/27 °C L/D) in a 12/12 h L/D cycle at the same PPFD. All measurements were made at the end of the second phase, at the stem-elongation stage, when the leaf sheaths had become erect. Each temperature treatment was applied in a separate growth chamber and repeated three times.

### 2.2 Leaf gas exchange analysis

The third fully developed leaf of each seedling was used for foliar gas-exchange analysis. Gas exchange analysis was measured using a CIRAS 3 Portable Photosynthesis System (PP Systems, Amesbury, MA, USA) with a narrow leaf chamber (1.75 cm^2^). Measurements were made at a common laboratory temperature; however, the treatment temperatures (20 °C, 35 °C, and 40 °C) and an atmospheric CO_2_ concentration of 400 µL L^−1^ were set inside the IRGA cuvette, and an actinic light intensity of 250 µmol m^−2^ s^−1^ was provided by the blue (*λ* = 450 nm) LED array. The net photosynthetic rate (*P_n_*), stomatal conductance (*g_s_*), transpiration rate (*E*), and water-use efficiency (WUE) were recorded once photosynthesis had reached steady state (after 15 min). Five leaves, each from a different plant grown in a separate pot, were analysed.

### 2.3 Photosynthetic pigment measurements

The total anthocyanin content was quantified using Neff and Chory’s (1998) method. Two hundred milligrams of the central parts of the third fully developed leaves were ground in 0.7 mL of 60% (v/v) ice-cold methanol-HCl, followed by overnight incubation in the cold and dark. Crude extracts were centrifuged at 12,000 × *g* for 10 min and kept in the cold and dark. Anthocyanin forms were separated with chloroform, and their content was determined by measuring absorbance at 530 and 657 nm and calculating A530 − (0.25 × A657), using *ϵ* = 30 mM^−1^ cm^−1^ at 530 nm. The anthocyanin content is expressed as nanomoles per gramme of leaf fresh weight. Fifty milligrammes of the central portions of the third fully developed leaves were ground in 4 mL of 80% (v/v) ice-cold acetone to determine total chlorophyll and carotenoid contents. The extracts were centrifuged at 12,000 × *g* for 10 min and kept at 0 °C in the dark before the measurement. Absorbance was measured at 664, 646, and 470 nm using a UV-VIS 160A spectrophotometer (Shimadzu, Kyoto, Japan). Pigment contents were calculated using Lichtenthaler’s (1987) method, as follows: CHL*_a_* = 12.24 A_664_ − 2.79 A_646_, CHL*_b_* = 21.5A_646_ − 5.1 A_664_, CHL*_a_*_+_ *_b_* = 7.15A_664_ + 18.71 A_646_ and Xanthophylls + carotenoids = (1,000 A470 − 1.82 CHL*_a_* − 85.02 CHL*_b_*)/198. The chlorophyll and carotenoid contents are expressed as microgrammes per gramme of leaf fresh weight.

### 2.4 Enzyme activity assays

#### 2.4.1 Analysis of enzyme activity of GR, GST, APX, and DHAR

Fully developed leaves (0.3 g) were homogenised in ice-cold 100 mM Tris-HCl buffer (pH 7.5), containing 3 mM MgCl_2_ and 0.5 mM EDTA, and centrifuged at 10,000 × *g* for 10 min. The samples were kept on ice prior to measurement. The spectrophotometric assays were performed at room temperature. APX activity was measured in a Tris-HCl buffer (0.2 mM, pH 7.8) containing 25 mM ascorbic acid and 0.5 mM H_2_O_2_. The total volume of the reaction mixture was 2.25 mL; 50 µL of plant sample was added, and the reaction was initiated by adding the H_2_O_2_ solution. Decay of ascorbic acid was monitored at 290 nm (*ϵ* = 2.8 mM^−1^ cm^−1^) (Nakano and Asada, 1981). GR activity was determined from the reduction of 5,5′-dithio-bis-(2-nitrobenzoic acid) at 412 nm (*ϵ* = 14.15 mM^−1^ cm^−1^) according to the method of Smith et al. (1988). The reaction mixture contained 100 mM sodium phosphate buffer (pH = 7.5), 1 mM EDTA, 0.75 mM DTNB, 0.1 mM NADPH, and 1 mM GSSG. The 1-mL reaction mixture contained 50 µL of plant sample. GST activity was determined in a mixture of 72.7 mM sodium phosphate buffer (pH 6.5), 3.6 mM reduced glutathione (GSH), 1 mM 1-chloro-2,4-dinitrobenzene, and crude enzyme extract (100 µL of plant sample in 2.75 mL of reaction mixture). Changes in absorbance were recorded at 340 nm (*ϵ* = 9.6 mM^−1^ cm^−1^) (Mannervik and Guthenberg, 1981). For the DHAR assay, 400 mg of leaves were homogenised, and 700 µL of saturated ammonium sulphate was added to inhibit APX activity. After 10 min, the homogenate was centrifuged at 12,000 × *g* for 10 min. The reaction mixture contained 2 mL of 100 mM sodium phosphate buffer (pH 7.5), 300 µL of 10 mM dehydroascorbic acid, and 300 µL of 2.5 mM GSH solution. The reaction was initiated by adding 100 µL of enzyme extract. The increase in absorbance resulting from GSH-dependent ascorbic acid production was monitored at 265 nm (*ϵ* = 14.5 mM^−1^ cm^−1^) (Hossain and Asada, 1984).

#### 2.4.2 Enzyme activity assays of AKR, AOR/AER, and GLY I

The third fully developed leaves (0.3 g) were homogenised in 900 µL of cold 50 mM sodium phosphate buffer solution (pH 7.0). The homogenates were centrifuged at 10,000 × *g* for 10 min and then filtered through 10 kDa Ultracel filters (Merck Millipore, Burlington, MA, United States) for 60 min. Prior to spectrophotometric analysis, all samples were stored on ice. AKR and AOR/AER activities were measured in the same assay, which contained filtered tissue extract and 50 mM sodium phosphate buffer in a a:9 ratio (pH 7.0). To initiate the reactions, 20 mM solutions of propionaldehyde and 3-buten-2-one were added. NADPH was also added to a final concentration of 0.1 mM. Changes in absorbance were monitored at 340 nm (*ϵ* = 6.22 mM^−1^ cm^−1^) (Yamauchi et al., 2011). The GLY I-specific assay contained 100 mM sodium phosphate buffer (pH 7.5), 3.5 mM methylglyoxal, 1.7 mM reduced glutathione, and 16 mM MgSO_4_. Following a modified method of Johansen et al. (2000), filtered extracts were added at a ratio of 1:9 to bring the final assay volume to 1 mL. The formation of the glutathione esters (*ϵ* = 3.37 mM^−1^ cm^−1^) was monitored at 240 nm.

#### 2.4.3 PAL assay

The PAL activity was measured according to Gao et al. (2008) using 1 g of leaves homogenised with 4 mL of 50 mM Tris-HCl buffer (pH 8.8, containing 5 mM β-mercaptoethanol and 4% (v/v) polyvinylpyrrolidone). After centrifugation (10 min, 10,000 × *g*), enzyme activity was determined using 250 µL of supernatant in a total reaction volume of 3 mL containing 50 mM phosphate buffer (pH 8.8) and 50 mM phenylalanine. The reaction was stopped with 10% (v/v) trichloroacetic acid after a 1-h incubation at 37 °C, and the *trans*-cinnamic acid produced was detected spectrophotometrically at 290 nm.

All spectrophotometric enzyme activity assays were performed at room temperature using a Shimadzu UV-VIS 160A (Shimadzu, Kyoto, Japan). Enzyme activities were calculated and expressed in nanokatals per gramme of leaf fresh weight.

### 2.5 Polyamine and DAP analysis

The analysis was carried out according to the method described by Németh et al. (2002). The polyamines Put, cadaverine (CAD), Spd, and Spm, as well as DAP (the product of terminal catabolism of Spd and Spm), were analysed as dansylated derivatives via HPLC. This was done using a W2690 separation module on a reversed-phase column (Kinetex C18, 5 µm, 100 mm × 4.6 mm, Phenomenex, Torrance, CA, United States) and a W474 scanning fluorescence detector with excitation at 340 nm and emission at 515 nm (Waters, Milford, MA, USA). Polyamine and DAP contents were quantified against external standards and expressed as microgrammes per gramme of leaf fresh weight.

### 2.6 Statistical analysis

All analyses used R 4.4.0 (R Core Team, 2024), with figures drawn in ggplot2 (Wickham, 2016). The independent biological replicates per genotype × temperature combination ranged from three to seven depending on the parameter, and the exact number for every parameter and treatment combination is given in the deposited data. Replicate numbers, means, standard deviations, standard errors, and Tukey letters of every trait are given in **Supplementary Table S1**, while the ANOVA results are given in **Supplementary Table S2**. Each trait was fitted separately to a two-way factorial linear model with genotype, temperature, and their interaction as fixed effects (Equation 1):

(1) $$y_{ijk}=\mu +G_{i}+T_{j}+\;{\left(G\times T\right)}_{ij}+\epsilon_{ijk}$$

where *y_ijk_* is the observation for plant *k* of genotype *i* (six levels) at temperature *j* (three levels: 20 °C, 35 °C, and 40 °C). Both factors were unordered and used treatment contrasts, with Mv Tarsoly and 20 °C as the reference levels. *G_i_*, *T_j_*, and (*G* × *T*)*_ij_* are the fixed effects of genotype, temperature, and their interaction, and *ϵ_ijk_* is an independent normally distributed error. Main effects and the interaction were tested by analysis of variance with Type II sums of squares (car; Fox and Weisberg, 2019). Residual normality (Shapiro–Wilk) and variance homogeneity across the 18 genotype × temperature groups (Levene) were checked for each trait on the raw scale. Where either failed, the trait was reanalysed after a power transformation (natural logarithm or square root) that satisfied or improved both diagnostics. Shoot biomass, DHAR, GR and 1,3-diaminopropane were log-transformed. The four gas-exchange and four pigment traits were also log-transformed, one transformation per group, so that the gas-exchange panels and the pigment panels each share a scale. GST, AOR/AER, glyoxalase I, spermine, spermidine, cadaverine, and putrescine were square-root-transformed; APX, AKR, and PAL were untransformed. Water-use efficiency, anthocyanin, and putrescine still deviated from normality on the chosen scale but were retained, the *F*-test being robust to such departures at these group sizes. Diagnostics for every trait on both scales accompany the code.

Given that the genotype × temperature interaction was significant for most traits, cultivars were compared within temperatures. The 15 pairwise comparisons among the six cultivars at each temperature were based on estimated marginal means from the fitted model (emmeans; Lenth, 2024), on the transformed scale where applicable. *p*-values were Tukey-adjusted (Tukey, 1949) and summarised as compact letter displays (multcomp; Hothorn et al., 2008); letters were assigned in descending order of the means, so that cultivars not sharing a letter within a temperature differed at *p* < 0.05. Differences between temperatures within a cultivar are reported in the Results as percentage changes from that cultivar’s 20 °C control.

Trait relationships and overall response patterns were examined by multivariate analysis of the genotype × temperature means. The three temperatures were analysed together because the aim was to describe how the trait profile of each cultivar shifts with temperature; treatment identity is retained in the ordination by colour, label and a separate ellipse for each temperature. Principal component analysis (PCA) was performed using the prcomp function on 21 traits centred and scaled to unit variance, which is equivalent to using their correlation matrix. Vapour-pressure deficit was excluded because it was collinear with temperature, whereas anthocyanin was excluded because it was measured only at 20 °C and 40 °C; excluding these variables allowed all 18 observations to be retained without missing values. The variance explained by each component was calculated from the eigenvalues, and the first two components were represented in a biplot of sample scores and trait loadings, with 68% normal-probability ellipses for each temperature. Pairwise associations among the 22 traits (excluding vapour-pressure deficit) were quantified across the 18 genotype × temperature means using Spearman’s rank correlation based on pairwise-complete observations (12 observations for anthocyanin). Significance was assessed using cor.test, and only coefficients with *p* < 0.05 are displayed (corrplot; Wei and Simko, 2021). These pooled coefficients mainly describe the response to temperature shared by all cultivars (**Supplementary Figure S1**). To isolate covariation among cultivars, the correlations were recomputed after centring each trait within temperature, that is, after subtracting the mean of the six cultivars at that temperature. The *p*-values were calculated using the *t* approximation with *N* − *k* − 1 degrees of freedom, where *k* is the number of temperatures (**Supplementary Figure S2**).

Trait maintenance under stress was quantified by a trait-specific stress-tolerance index (STI) adapted from the yield-based index of Fernandez (1992), computed from genotype × temperature means (Equation 2):

(2) $$\text{STI }=\;\left(Y_{p}\times Y_{s}\right)/{\left({\bar{Y}}_{p}\right)}^{2}$$

where *Y_p_* and *Y_s_* are the control (20 °C) and stressed (35 °C or 40 °C) values of a trait in a given cultivar, and *Ȳ_p_* is its mean control value across all six cultivars. The STI was computed for every cultivar at each stress level for the 21 traits retained in the PCA, giving a 12 × 21 matrix (six cultivars × two stress levels). Within each trait, values were standardised to *Z*-scores (mean 0, SD 1 across the 12 values) and shown as a heatmap (ComplexHeatmap; Gu et al., 2016). Columns were ordered by hierarchical clustering on Euclidean distances with Ward’s minimum-variance criterion (Ward, 1963); rows were split by stress level (35 °C versus 40 °C) and clustered within each block. Each stressed value is scaled by both its own control and the panel-wide control mean; therefore, a high index indicates that a trait was better maintained than in the other cultivars. For traits that increase under heat, such as the three diamines, a high index instead means a larger stress-induced increase, so the diamine columns read in the opposite direction to the pigment, antioxidant, and carbonyl-detoxification columns. Cultivars are classified as heat-tolerant, intermediate, or heat-sensitive according to a single criterion: their combined position in the PCA and the trait-wise STI, both computed across all retained traits and reported in Multivariate and stress tolerance index analyses resolve the tolerance ranking; no individual trait was used. On this basis, Mv Lucilla, Mv Kolompos, and Mv Dandár form the tolerant group, Mv Pirkadat forms the intermediate group, and Mv Ikva and Mv Tarsoly form the sensitive group. The raw data and the R scripts that reproduce all analyses and figures of this study are available at Zenodo (https://doi.org/10.5281/zenodo.22692577) and GitHub (https://github.com/yawarhabib/Genotype-specific-heat-responses-in-winter-wheat).

## 3 Results

### 3.1 Shoot biomass declines with heat in every cultivar

Shoot fresh weight decreased in a temperature-dependent manner in every cultivar, being highest at 20 °C, intermediate at 35 °C, and lowest at 40 °C (**Figure 1**). Moderate heat already reduced biomass relative to the control, and severe heat reduced it further in all genotypes, but the magnitude of the loss was strongly genotype-dependent. Relative to their own controls, Mv Dandár retained the most biomass at 40 °C (23% loss), whereas Mv Kolompos retained the least (66%); Mv Tarsoly, Mv Ikva, Mv Lucilla, and Mv Pirkadat showed intermediate losses (38%–58%). This ordering largely reflects differences in control vigour: Mv Kolompos and Mv Pirkadat had the highest control biomass, 1.8–2.0 times that of Mv Ikva and Mv Dandár, and the range of absolute biomass narrowed markedly at 40 °C, so that proportional loss favours the less vigorous genotypes. Mv Kolompos accordingly moved from the highest group under control conditions to the lowest at 40 °C, so absolute biomass under severe heat did not follow control vigour.

**Figure 1:**
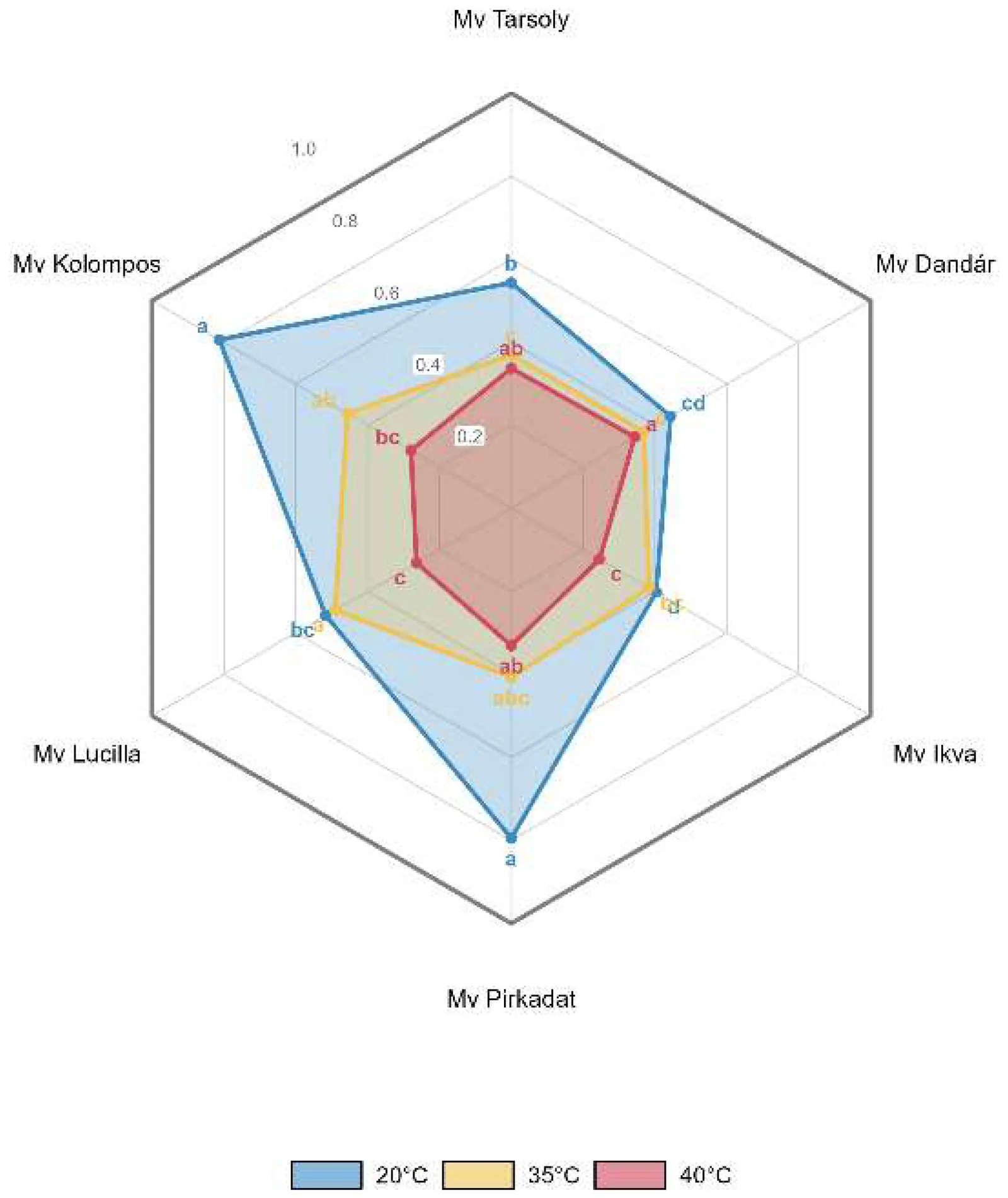
Shoot fresh weight of six Mv winter wheat cultivars at 20°C (control), 35°C, and 40°C. Values are shoot fresh weight (g per plant). Letters compare cultivars within a temperature (Tukey HSD, p < 0.05).

### 3.2 Gas exchange is impaired, and water-use efficiency declines steeply under heat, uniformly across cultivars

Both *P_n_*, *g_s_*, and WUE were highest under control conditions and declined progressively with temperature, with the reductions being most pronounced at 40°C (**Figure 2**). *E*, by contrast, increased under heat, peaking at 35°C, reflecting the steeper leaf-to-air vapour-pressure gradient that accompanies heat; the combination of decreasing *P_n_* and increasing *E* produced a pronounced reduction in WUE under severe heat in all cultivars. Only Mv Pirkadat, which had the lowest cultivar throughout, differed significantly from the highest-ranking cultivar in *P_n_*, *g_s_*, and *E*, and the groupings did not change with temperature; the two differences in WUE observed under control conditions were no longer significant under heat.

**Figure 2:**
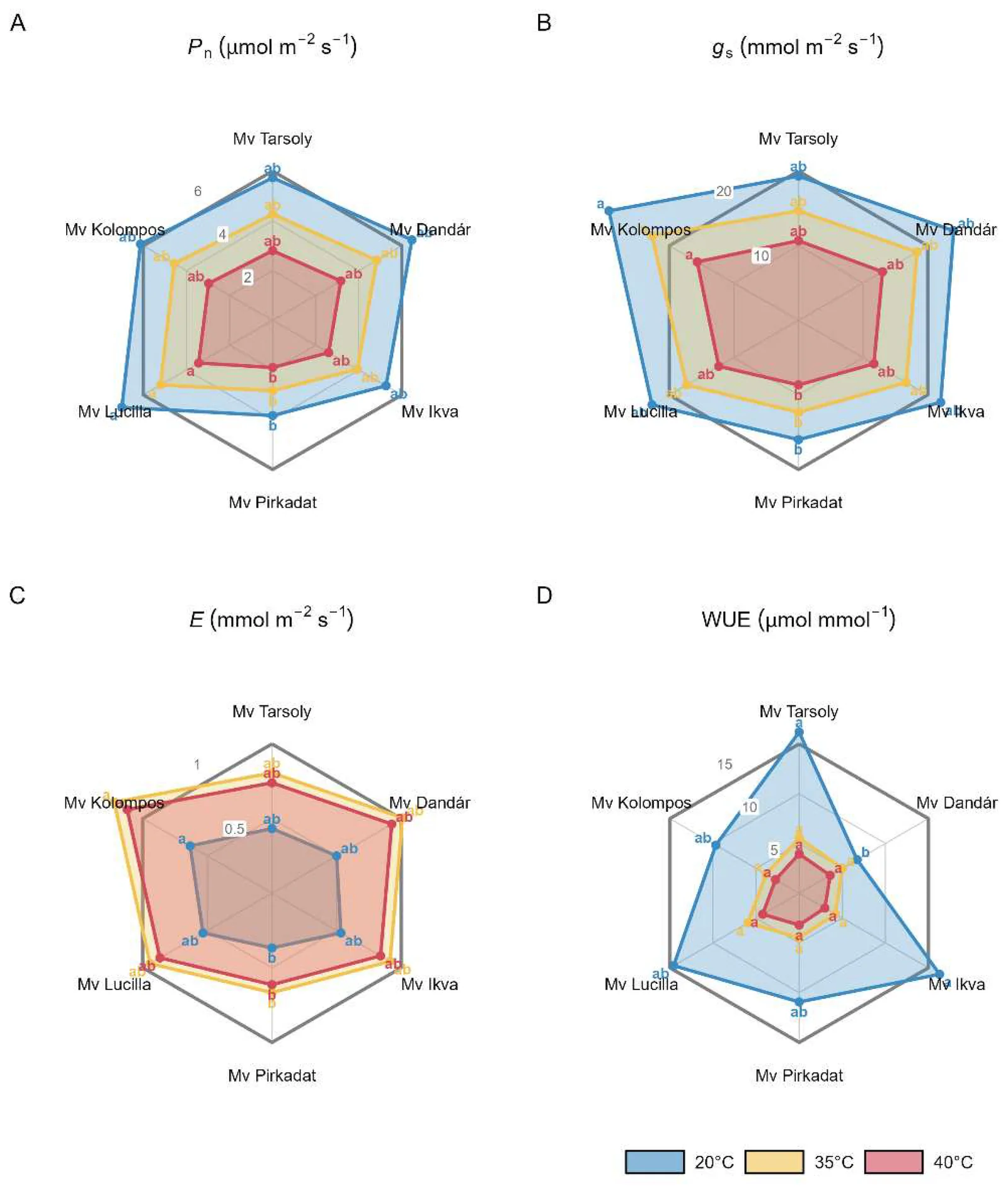
Leaf gas exchange parameters—net photosynthesis rate (P_n_) **(A)**, stomatal conductance (g_s_) **(B)**, transpiration rate (E) **(C)** and water-use efficiency (WUE) **(D)**—of the six wheat cultivars at 20°C, 35°C, and 40°C. Letters compare cultivars within a temperature (Tukey HSD, p < 0.05).

### 3.3 Pigment loss under severe heat differs between cultivars

Total chlorophyll (Chl *a*+*b*) declined steadily with temperature in all cultivars (**Figure 3**). At 40 °C, the loss was most severe in Mv Tarsoly (49% below control) and Mv Pirkadat (39%), intermediate in Mv Ikva and Mv Dandár (28%–30%), and smallest in Mv Kolompos (22%) and Mv Lucilla (20%), which retained the highest chlorophyll content under severe heat, although only Mv Lucilla, Mv Kolompos, and Mv Ikva differed significantly from Mv Tarsoly in absolute chlorophyll content at 40 °C. The chlorophyll *a*/*b* ratio followed the same pattern, declining least in Mv Lucilla and Mv Kolompos (14%–15%) and most in Mv Pirkadat and Mv Tarsoly (33%–34%) at 40 °C. The *a*/*b* ratio separated the cultivars more sharply than total chlorophyll, with Mv Lucilla highest and Mv Pirkadat and Mv Tarsoly lowest at 40 °C. In contrast to chlorophyll, carotenoids increased at 35 °C in all cultivars and were largely sustained at 40 °C in the tolerant genotypes; Mv Kolompos and Mv Lucilla maintained or slightly exceeded their control levels (+ 3%), whereas carotenoid levels declined by 19% in Mv Tarsoly and by 16% (below control) in Mv Pirkadat. Anthocyanins accumulated strongly under severe heat, increasing several-fold at 40 °C. The increase was steepest in Mv Lucilla (4.4-fold) and Mv Ikva (3.0-fold), whereas the highest absolute levels were observed in Mv Pirkadat and Mv Lucilla. Increases were more modest in the other cultivars, while Mv Kolompos, which already had a high constitutive level, showed essentially no further increase. The cultivars did not differ significantly in anthocyanin content at either temperature.

**Figure 3:**
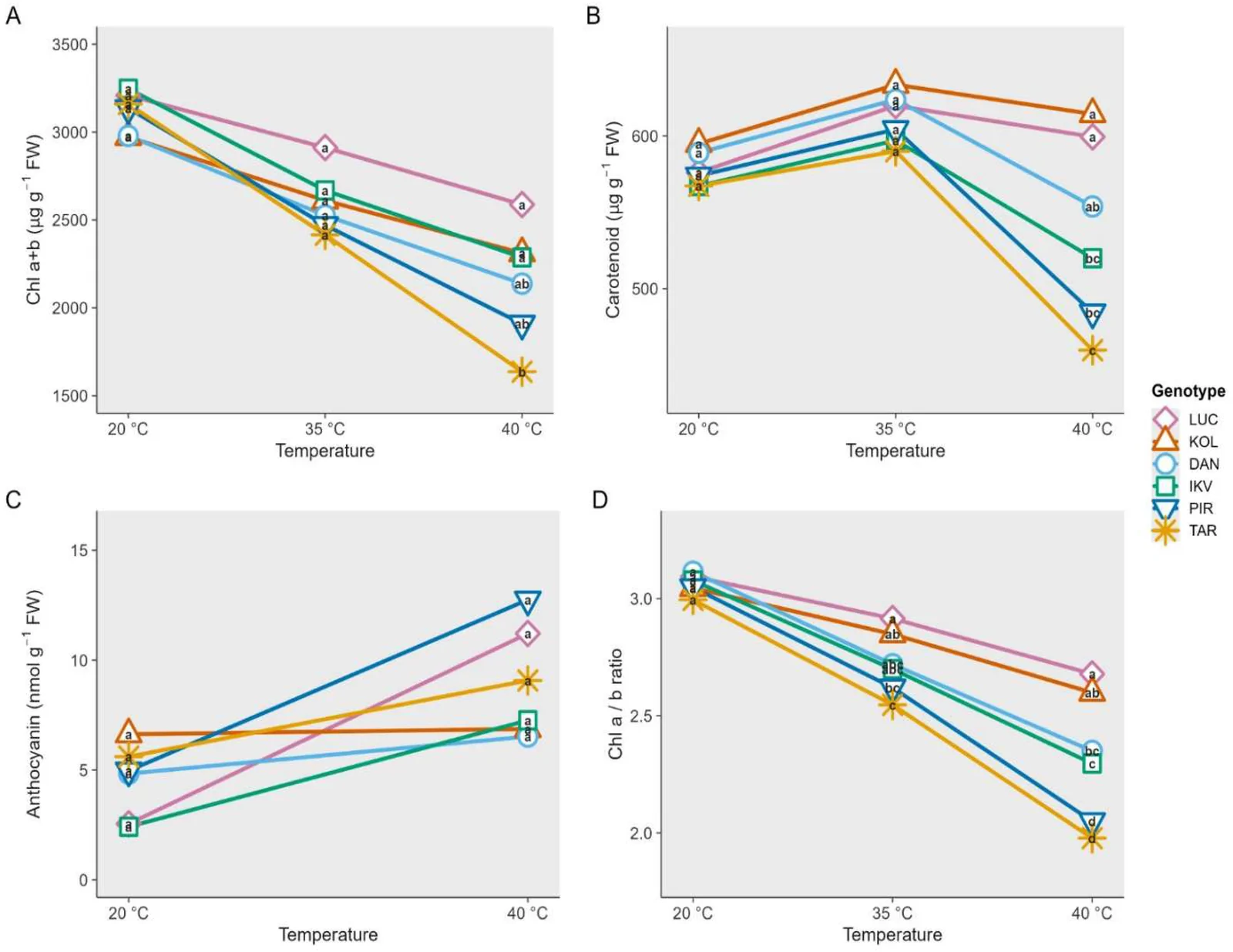
Photosynthetic pigments of the six cultivars across the three temperatures: **(A)** total chlorophyll content (Chl a + b), **(B)** carotenoids, **(C)** anthocyanins, and **(D)** the chlorophyll a/b ratio. LUC, Mv Lucilla; KOL, Mv Kolompos; DAN, Mv Dandár; IKV, Mv Ikva; PIR, Mv Pirkadat; TAR, Mv Tarsoly. Letters compare cultivars within a temperature (Tukey HSD, p < 0.05).

### 3.4 The AsA–GSH antioxidant enzymes show a coordinated, genotype-specific response

Antioxidant enzyme activities responded in a clearly dose-dependent manner, with most activities increasing at 35 °C and then diverging among cultivars at 40 °C (**Figure 4**). APX activity (**Figure 4A**) was upregulated under heat in five of the six cultivars; the response was strongest in those with low constitutive activity, with activity increasing roughly threefold at 40 °C in Mv Pirkadat and Mv Ikva and nearly doubling in Mv Dandár, whereas Mv Tarsoly was the only cultivar showing APX activity below the control level at 40 °C (24% lower), while Mv Kolompos had the highest absolute APX activity at 40 °C. DHAR activity (**Figure 4B**) peaked at 35 °C in most cultivars and then declined sharply at 40 °C, to levels roughly 30%–50% below control in Mv Tarsoly, Mv Ikva, Mv Dandár, and Mv Pirkadat. Mv Kolompos was the notable exception, maintaining a statistically unchanged DHAR activity across all three temperatures. It was the only cultivar in which severe heat did not compromise dehydroascorbate reduction, and its DHAR activity at 40 °C exceeded that of every other cultivar. GR activity (**Figure 4C**) increased with temperature in every cultivar and remained elevated at 40 °C, with the largest increases occurring in Mv Ikva (85%) and Mv Kolompos (59%), so that glutathione regeneration was broadly maintained even under severe stress; these percentages represent proportional gains from different baselines; in absolute terms, Mv Kolompos had the lowest GR activity of the six cultivars at 40 °C. GST activity (**Figure 4D**) peaked at 35 °C and, at 40 °C, was maintained at or near control levels in the tolerant cultivars Mv Kolompos and Mv Dandár but declined in the sensitive cultivars Mv Ikva (27%) and Mv Tarsoly (20%), while Mv Kolompos showed the highest absolute GST activity at 40 °C. Taken together, the tolerant cultivars sustained a broad and stable antioxidant response under severe heat, exemplified by Mv Kolompos, which uniquely preserved both DHAR and GST while increasing APX and GR, whereas Mv Tarsoly displayed the weakest profile, with APX, DHAR, and GST all decreasing below their control values.

**Figure 4:**
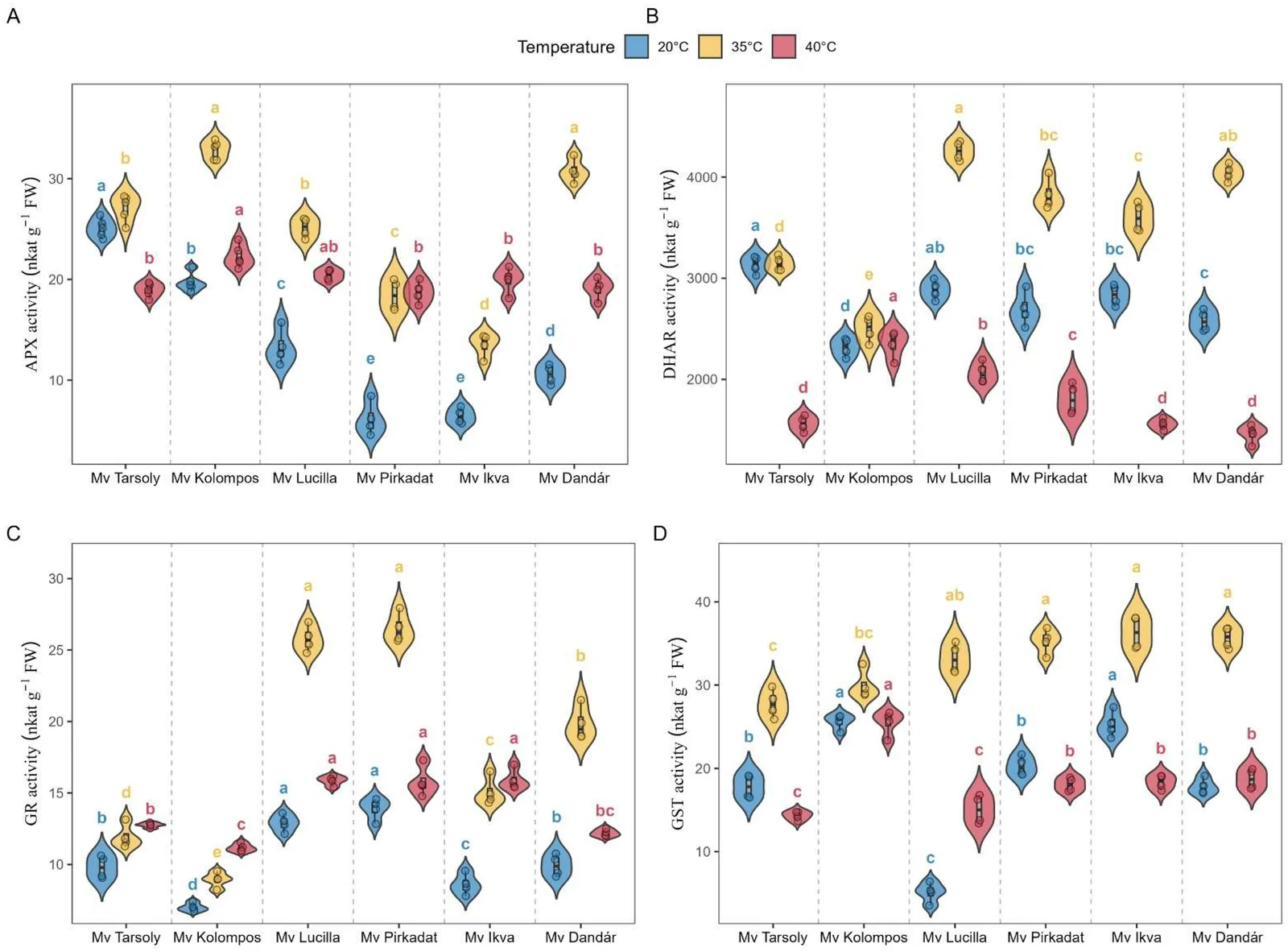
The activities of the enzymes of the ascorbate–glutathione cycle and glutathione S-transferase in the six wheat cultivars at 20°C, 35°C, and 40°C. **(A)** Ascorbate peroxidase (APX), **(B)** dehydroascorbate reductase (DHAR), **(C)** glutathione reductase (GR), and **(D)** glutathione S-transferase (GST). Letters compare cultivars within a temperature (Tukey HSD, p < 0.05).

### 3.5 Reactive-carbonyl detoxification is sustained in tolerant cultivars and declines in Mv Tarsoly

The reactive-carbonyl detoxification enzymes were strongly induced at 35 °C in every cultivar, indicating a vigorous activation of the carbonyl-detoxification machinery under moderate heat (**Figure 5**). Glyoxalase I (**Figure 5C**) increased three- to nearly five-fold at 35 °C (between + 243% in Mv Ikva and + 366% in Mv Lucilla), and AOR/AER (**Figure 5B**) increased by 140–244%. The critical distinction emerged at 40 °C. Glyoxalase I remained well above control in the tolerant and intermediate cultivars (by approximately + 245% in Mv Kolompos, + 203% in Mv Dandár, + 179% in Mv Pirkadat, + 160% in Mv Lucilla, and + 126% in Mv Ikva), whereas in Mv Tarsoly it decreased below the control level (− 8%), significantly lower than in the other five cultivars; the same pattern was observed for AOR/AER. AKR activity (**Figure 5A**), by contrast, peaked at 35 °C and then declined below control at 40 °C in all cultivars except Mv Kolompos, which maintained it essentially at its control level (+ 7%); the reductions ranged from 24% in Mv Pirkadat and 27% in Mv Dandár to 49% in Mv Tarsoly. The accompanying heatmap (**Figure 5D**) summarises these patterns and shows that Mv Dandár and Mv Kolompos retained some of the highest overall levels of carbonyl-detoxification activity under severe heat, while Mv Tarsoly was the only cultivar in which both glyoxalase I and AOR/AER were suppressed below their control values.

**Figure 5:**
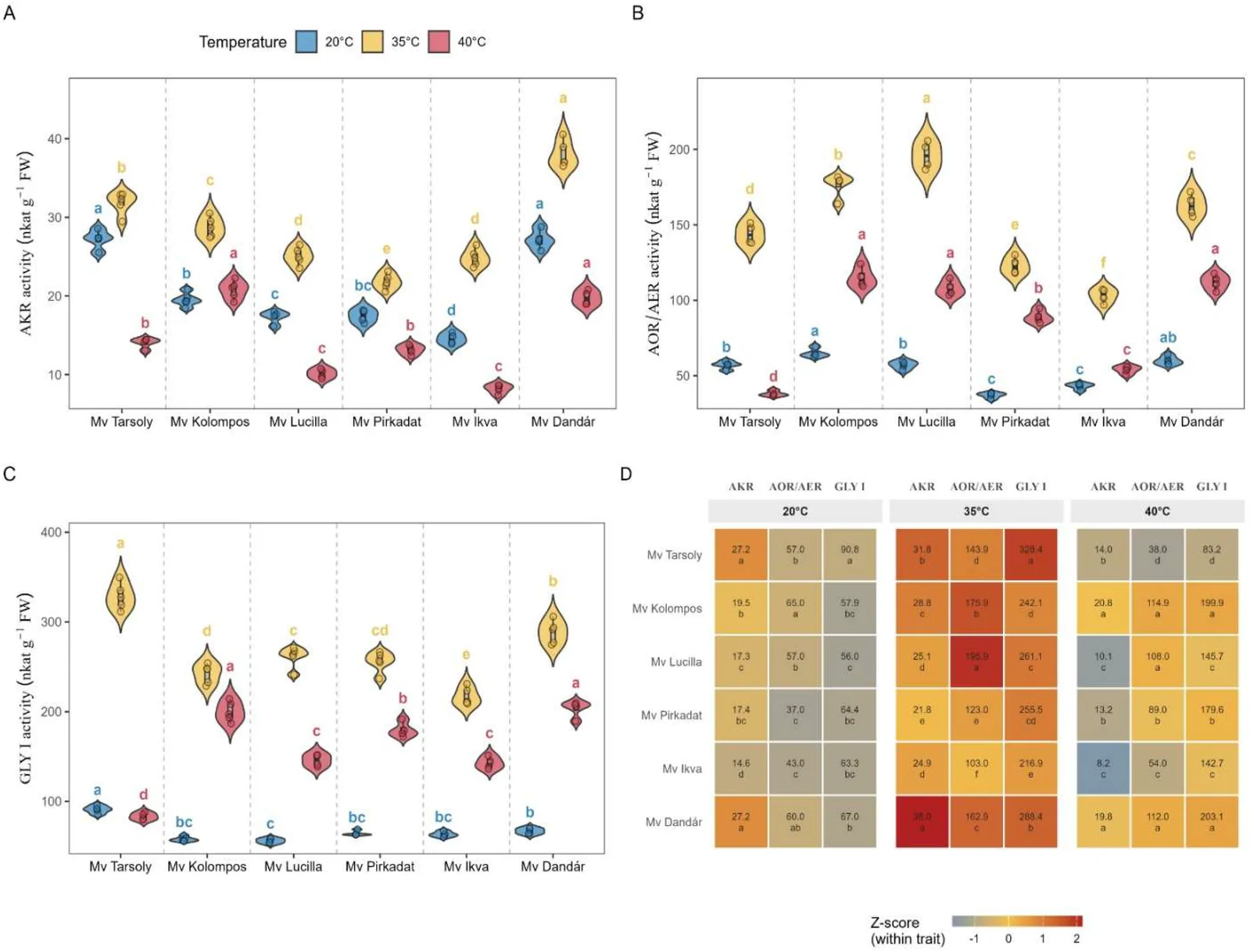
Changes in the activities of the reactive-carbonyl detoxification enzymes in the six cultivars. **(A)** aldo-keto reductase (AKR), **(B)** alkenal/alkenone oxidoreductase (AOR/AER), and **(C)** glyoxalase I (GLY I) at 20°C, 35°C, and 40°C, respectively; **(D)** heatmap of mean activities (within-trait Z-scores). Letters compare cultivars within a temperature (Tukey HSD, p < 0.05).

### 3.6 PAL activity peaks at moderate heat and diverges under severe stress

PAL activity increased at 35 °C in all cultivars and then diverged at 40 °C (**Figure 6**). It was sustained at or above control in Mv Tarsoly (+ 28%), Mv Kolompos (+ 16%), and Mv Ikva (+ 4%), but declined in Mv Lucilla (33%) and Mv Pirkadat (19%) and decreased steeply in Mv Dandár (56% below control), which had the lowest PAL activity among the six cultivars, with a value significantly below those of the other cultivars, whereas Mv Tarsoly had the highest.

**Figure 6:**
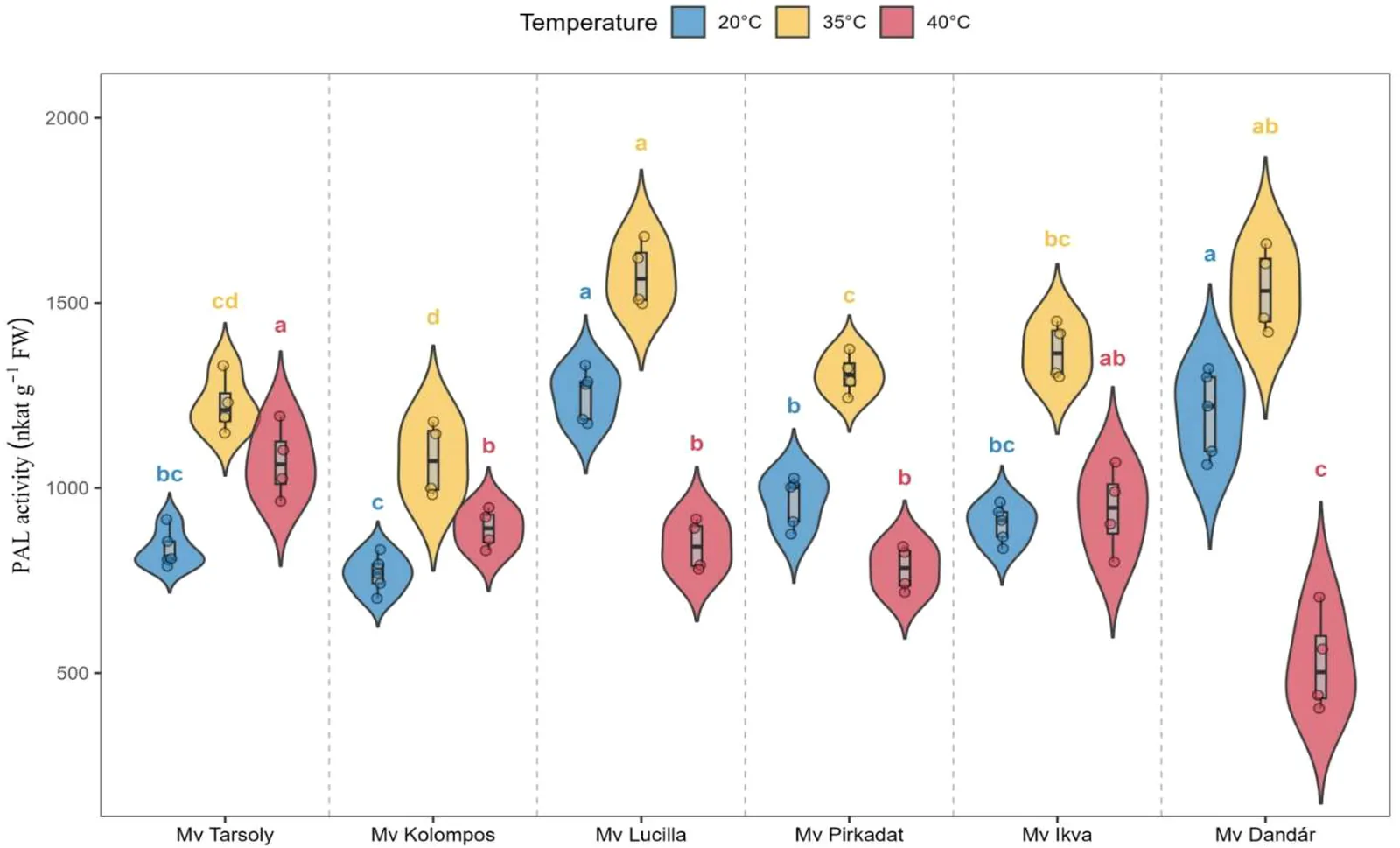
Phenylalanine ammonia-lyase (PAL) activity of the six cultivars at 20°C, 35°C, and 40°C. Letters compare cultivars within a temperature (Tukey HSD, p < 0.05).

### 3.7 Polyamine pools shift from higher polyamines to catabolic diamines under heat

Heat stress extensively reconfigured the polyamine pools, and the direction of change differed sharply between the higher polyamines and the diamines (**Figure 7**). The higher polyamines Spm and Spd decreased with temperature in almost every cultivar. At 40 °C relative to the control, Spm decreased by roughly 58%–77% and Spd by roughly 31%–57%; the exception was Mv Kolompos, in which Spd recovered to slightly above its control level. In contrast, the diamines Put, Cad, and DAP accumulated several-fold under heat at 40 °C, with DAP increasing markedly in all genotypes. The accumulation of the two stress-associated diamines was strongly genotype-dependent. At 40 °C, Put was highest in Mv Ikva, followed by Mv Pirkadat and Mv Kolompos, whereas Cad was highest in Mv Tarsoly, which showed the largest accumulation among the cultivars, followed by Mv Ikva and Mv Lucilla; in contrast, Mv Dandár accumulated comparatively little of either diamine, while Mv Lucilla had the lowest Put and Mv Kolompos the lowest Cad. The two classes of polyamines also differed in when they distinguished among the cultivars: spermine, spermidine, and 1,3-diaminopropane separated the genotypes under control conditions but not at 40 °C, whereas cadaverine and putrescine did not distinguish among cultivars at 20 °C and 35 °C but did so under severe heat, with Mv Tarsoly reaching the highest cadaverine content and Mv Ikva the highest putrescine content.

**Figure 7:**
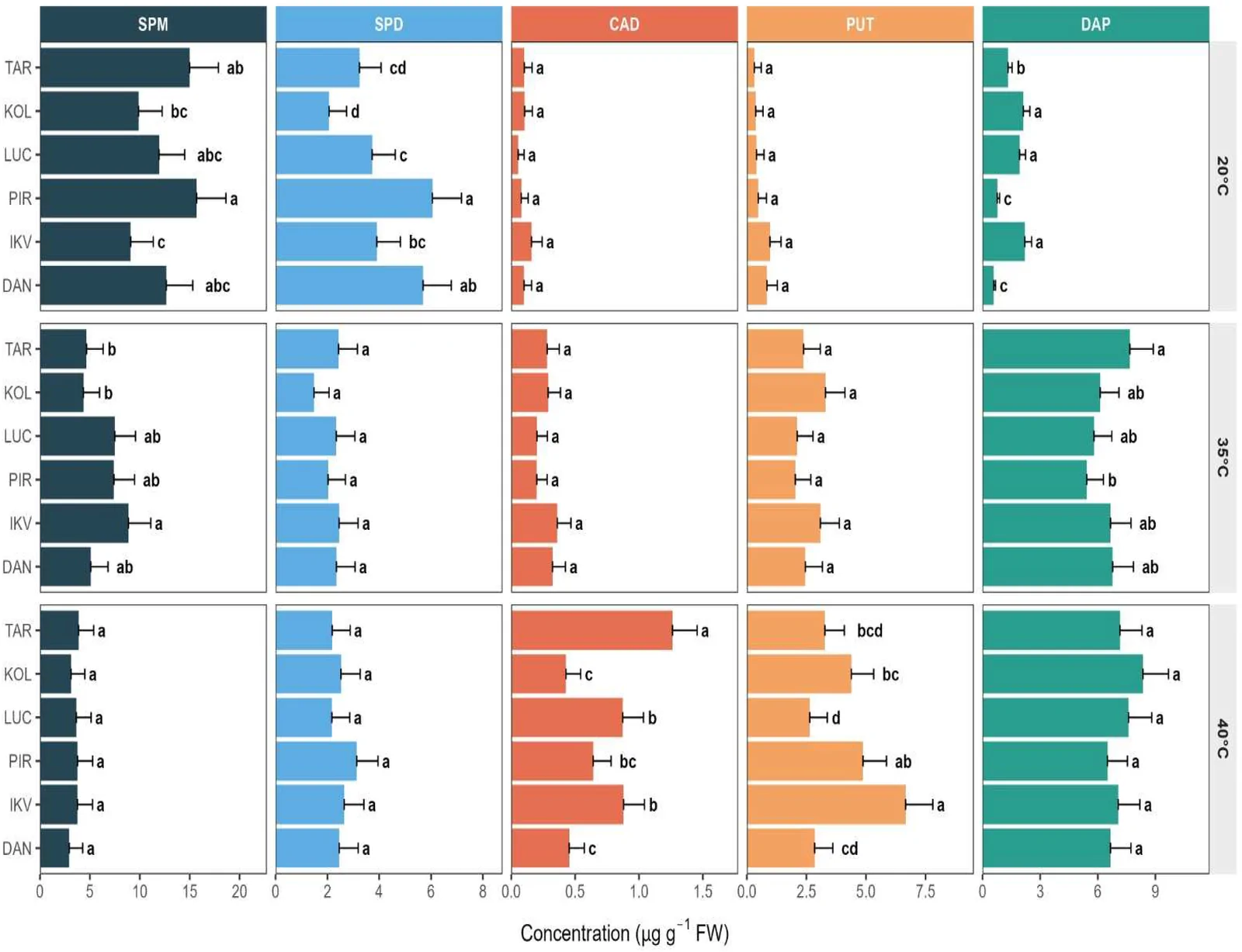
Polyamine pools—spermine (SPM), spermidine (SPD), cadaverine (CAD), putrescine (Put), and 1,3-diaminopropane (DAP)—in the six cultivars at 20°C, 35°C, and 40°C. Letters compare cultivars within a temperature (Tukey HSD, p < 0.05). Bars represent means + SE. LUC, Mv Lucilla; KOL, Mv Kolompos; DAN, Mv Dandár; IKV, Mv Ikva; PIR, Mv Pirkadat; TAR, Mv Tarsoly.

### 3.8 Multivariate and stress tolerance index analyses resolve the tolerance ranking

Principal component analysis integrated all measured traits and clearly separated the three temperature regimes (**Figure 8**; PC1 = 44.5% and PC2 = 28.0% of the variance, about 73% in total). PC1 separated the control samples, associated with high shoot biomass, chlorophyll, *P_n_*, *g_s_*, WUE, and spermine, from the heat-stressed samples, while PC2 distinguished the moderate (35 °C) treatment, aligned with the antioxidant and carbonyl-detoxification enzymes, carotenoids, and PAL, from the severe (40 °C) treatment, aligned with Put and Cad. Across the pooled means (**Supplementary Figure S1**), shoot biomass, *P_n_*, WUE, spermine, and chlorophyll were positively intercorrelated, and all were strongly negatively correlated with Put, Cad, and DAP, whereas carotenoids were not significantly correlated with this group and instead tracked the antioxidant and carbonyl-detoxification enzymes, most strongly with AOR/AER. Of 231 trait pairs, 100 were significant across the pooled means and 29 after centring within temperature (**Supplementary Figure S2**). Within each temperature, the strongest correlations were among gas exchange, carotenoids, the chlorophyll *a*/*b* ratio, and AOR/AER (*ρ* = 0.58 to 0.93). Carotenoids were correlated with AOR/AER in both analyses (*ρ* = 0.76 and 0.82), cadaverine was negatively correlated with glyoxalase I (*ρ* = − 0.60), and the diamines were not significantly correlated with shoot biomass, net photosynthesis, or chlorophyll. At the genotype level, the 40 °C samples of Mv Dandár, Mv Kolompos, and Mv Lucilla lay closest to the centre of the ordination; that is, they were the least displaced towards the severe-stress pole, whereas Mv Tarsoly, Mv Ikva, and Mv Pirkadat were the most extreme.

**Figure 8:**
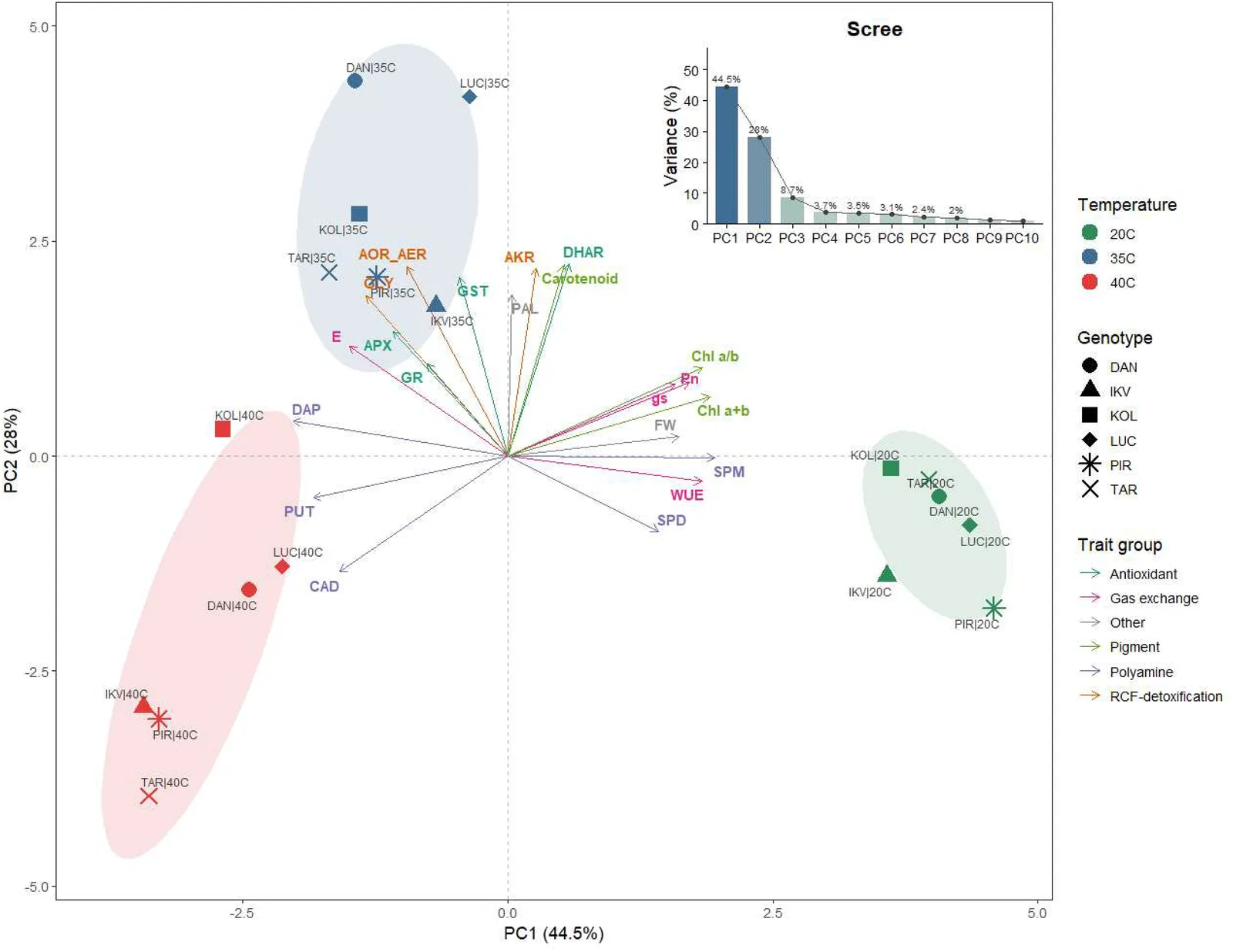
Principal component analysis (PCA) biplot of the measured traits, excluding anthocyanin, for six wheat cultivars at the three temperatures; the inset shows the variance explained by each component. Colours denote temperature. PCA was computed on standardised genotype × temperature means (vapour-pressure deficit and anthocyanin excluded). LUC, Mv Lucilla; KOL, Mv Kolompos; DAN, Mv Dandár; IKV, Mv Ikva; PIR, Mv Pirkadat; TAR, Mv Tarsoly.

In the trait-wise stress-tolerance index (**Figure 9**), displayed separately for the two stress levels, the trait clustering set the three diamines (putrescine, cadaverine, and 1,3-diaminopropane) apart from all of the other traits. At 40 °C, Mv Ikva and Mv Tarsoly showed the most extreme diamine indices, the highest cadaverine and, in Mv Ikva, by far the highest putrescine, together with the lowest AOR/AER, whereas Mv Kolompos and Mv Lucilla retained the highest carotenoid indices. The combined multivariate and index-based analysis ranked the cultivars from most to least heat-tolerant as follows: Mv Lucilla, Mv Kolompos, and Mv Dandár (which were statistically comparable), followed by Mv Pirkadat, Mv Ikva, and Mv Tarsoly.

**Figure 9:**
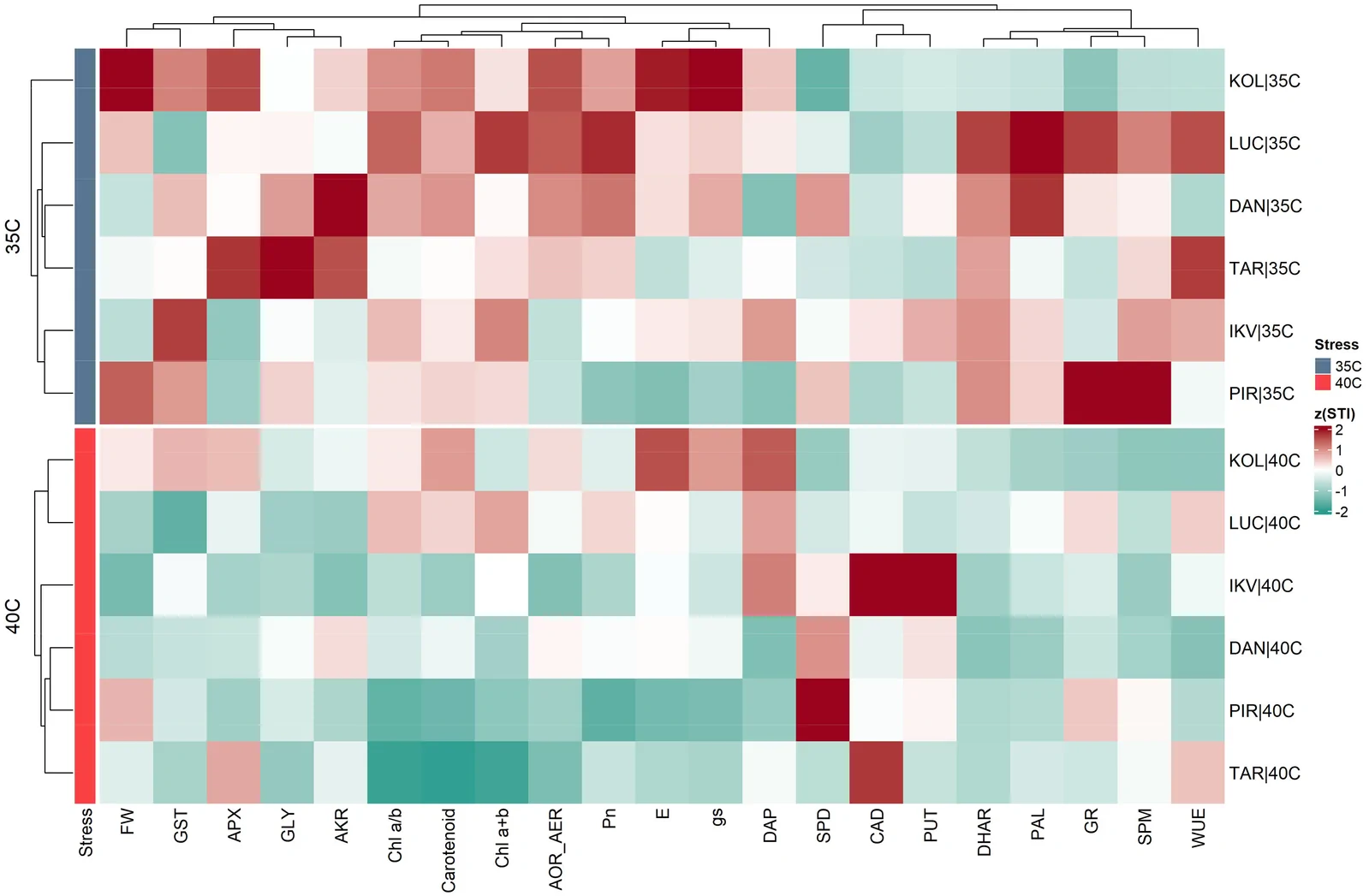
Stress-tolerance index (STI) heatmap of the six bread wheat cultivars at 35°C and 40°C, with rows grouped by stress level and hierarchically clustered within each level, while traits (columns) were also hierarchically clustered. STI follows Fernandez (1992); rows and columns were clustered by Ward’s method on Euclidean distances. LUC, Mv Lucilla; KOL, Mv Kolompos; DAN, Mv Dandár; IKV, Mv Ikva; PIR, Mv Pirkadat; TAR, Mv Tarsoly.

## 4 Discussion

Heat tolerance proved to be coordinated, dose-dependent, and genotype-specific. Two features stand out. First, the two regimes elicited qualitatively different responses: 35 °C broadly induced the protective enzymes, whereas cultivars separated decisively only at 40 °C, where the tolerant genotypes sustained that protection and the sensitive ones did not. Second, Mv Lucilla, Mv Kolompos, and Mv Dandár emerged as tolerant and Mv Tarsoly and Mv Ikva as the most sensitive, consistently across independent trait classes and analyses.

The progressive loss of biomass, chlorophyll, and net photosynthesis as temperature rises, alongside increased transpiration and a steep decline in water-use efficiency, is the expected signature of heat stress in wheat (Zheng et al., 2025; Lamba et al., 2023); recent work on contrasting wheat genotypes couples this decline to heat-shock proteins and the antioxidant system (Shenoda et al., 2026). The informative result is the genotypic spread: chlorophyll loss at 40 °C differed almost threefold between Mv Lucilla and Mv Tarsoly, and the cultivars retaining chlorophyll also preserved the highest *a*/*b* ratio. The *a*/*b* ratio is commonly taken to reflect light-harvesting antenna size, and its steeper decline in Mv Tarsoly and Mv Pirkadat is consistent with a disproportionate loss of photosystem II antenna pigments; however, net photosynthesis decreased similarly in every cultivar, so this pigment difference was not mirrored in Pn (Zheng et al., 2025; Sharma et al., 2015). The retention or slight increase in carotenoids in Mv Kolompos and Mv Lucilla is noteworthy: carotenoids quench singlet oxygen and dissipate excess excitation energy, limiting the oxidative load reaching the rest of the cell, while strong anthocyanin accumulation in Mv Lucilla and Mv Pirkadat adds a second screening and scavenging layer (Rahim et al., 2023; Mohi-Ud-Din et al., 2021).

The AsA–GSH enzymes and GST were induced at 35 °C in essentially all cultivars, reproducing the documented upregulation of these enzymes in heat-stressed wheat shoots (Goyal and Asthir, 2010; Iqbal et al., 2021; Janda et al., 2019). That the ascorbate–glutathione and glyoxalase systems are engaged together rather than separately is well established in wheat, where heat-tolerant varieties restrain both reactive oxygen species and methylglyoxal by jointly inducing both systems (Mohi-Ud-Din et al., 2022). Their behaviour at 40 °C was diagnostic: DHAR decreased in most cultivars but was unchanged in Mv Kolompos, GST remained at control levels in the tolerant cultivars but declined in Mv Tarsoly and Mv Ikva, and GR increased in every genotype while APX increased in all but Mv Tarsoly. The maintenance of APX and GR alongside the decrease in DHAR suggests that ascorbate regeneration, rather than peroxide scavenging, became the limiting step under severe heat (Iqbal et al., 2021). Recent work supports both aspects of this interpretation. Heat-induced methylglyoxal acts as a negative modulator of thermotolerance and impairs chloroplast protein import (Ding et al., 2026), so the carbonyl load is not merely a symptom of heat injury but a constraint on the heat response itself. The capacity to remove that load also varies within a species: *Arabidopsis* ecotypes differ in detoxifying glucose-derived reactive carbonyls, with the tolerant ecotype upregulating specific glyoxalase isoforms (Bouzid et al., 2025), and in related halophytes, the taxa that accumulated fewer reactive carbonyls recovered better (Patel et al., 2025). Glyoxalase activity is itself responsive to high-temperature stress and can be raised together with HSP90 by thiol supply (Durmuşoğlu et al., 2026). Our cultivars therefore differ in exactly the property these studies identify as limiting.

The reactive-carbonyl detoxification system showed a parallel pattern. Glyoxalase I and AOR/AER were strongly induced at 35 °C and remained well above control at 40 °C in every cultivar except Mv Tarsoly. Given that carbonyls such as methylglyoxal accumulate under heat and are removed mainly by the glyoxalase system (Hasanuzzaman et al., 2017; Rathore et al., 2024), this decline represents reduced carbonyl-detoxification capacity, while the activities sustained in Mv Dandár and Mv Kolompos are consistent with their tolerance. AKR declined at 40 °C in every cultivar except Mv Kolompos, as expected for an NADPH-dependent reductase (Yamauchi et al., 2011), suggesting that this branch is limited first when reducing power becomes scarce, so that in most cultivars, the glyoxalase pathway separates tolerant from sensitive genotypes. The link between this decline and heat sensitivity in Mv Tarsoly is correlative, but it rests on three indicators that are independent of one another. Mv Tarsoly was the only cultivar in which a carbonyl-detoxification arm decreased below control; both arms did so (glyoxalase I, − 8%; alkenal/alkenone oxidoreductase, − 33%); it had the lowest activities of both enzymes at 40 °C; and it accumulated significantly more cadaverine. It also ranked lowest in the multivariate and stress-tolerance-index analyses, which use all traits and are not weighted towards the carbonyl system. This convergence of independent assays on one cultivar is what supports the association; no causal role for either enzyme is claimed.

PAL behaved differently: it peaked at 35 °C in all cultivars, but was sustained at 40 °C in the lower-PAL genotypes (Mv Tarsoly, Mv Kolompos, Mv Ikva) and decreased in the high-PAL genotypes (Mv Lucilla, Mv Pirkadat, and Mv Dandár), particularly sharply in Mv Dandár. This single trait ran counter to the tolerance ranking, but is interpretable rather than contradictory. PAL diverts carbon from primary metabolism into the phenylpropanoid pathway (Rahim et al., 2023; Karamat et al., 2024). A plausible explanation is that this diversion becomes too costly at 40 °C, so it is reduced first in the genotypes with the highest activity, while lower activity can still be maintained. Similar treatment-dependent PAL responses have been reported in wheat (Gondor et al., 2016). That Mv Lucilla and Mv Dandár can downregulate PAL while remaining tolerant shows that tolerance in these cultivars arises from partly substitutable trait combinations rather than from one dominant mechanism.

Polyamine metabolism integrated the heat response most clearly. Across all cultivars, severe heat shifted the pool from the higher polyamines to the diamines: spermine decreased in every cultivar, and spermidine decreased in five of six, with Mv Kolompos as the exception; by contrast, putrescine, cadaverine, and 1,3-diaminopropane accumulated several-fold in all cultivars. This direction is explicable through polyamine catabolism: flavin-dependent PAO either back-convert spermine to spermidine and spermidine to putrescine or terminally cleave them to aminoaldehydes plus 1,3-diaminopropane, the terminal catabolite of the higher polyamines in wheat (Gondor et al., 2021), while copper-containing DAO oxidise putrescine; each reaction releases hydrogen peroxide, which doubles as a stress signal (Napieraj et al., 2023; Samanta et al., 2023; Pál et al., 2015, 2018; Rahman et al., 2024). The depletion of spermine, the increase in back-converted putrescine, and the strong accumulation of terminally derived 1,3-diaminopropane in all six cultivars are therefore the expected fingerprint of intensified catabolism, in agreement with other species (Upadhyay et al., 2020) and with wheat, where moderate heat lowered spermidine and spermine, left putrescine unchanged, and raised 1,3-diaminopropane in parallel with a polyamine-oxidase gene (Janda et al., 2019).

This remodelling differed by genotype in line with tolerance, more clearly at 40 °C than at 35 °C. The diamines accumulated most in the sensitive cultivars: putrescine was highest in Mv Ikva and cadaverine in Mv Tarsoly, whereas Mv Dandár and Mv Lucilla maintained both at low levels, and Mv Kolompos had the lowest cadaverine of the panel. Two recent findings frame this pattern. In alfalfa, endogenous spermidine and spermine were higher in the drought-resistant cultivar than in the sensitive cultivar and limited hydrogen peroxide production (Wang et al., 2023), the cultivar-level counterpart of the losses seen here. Cadaverine, by contrast, is not merely a passive marker: when applied exogenously, it alleviated salinity and bisphenol A toxicity in tomato (Abdelmoneim et al., 2025), so its accumulation in Mv Tarsoly is better interpreted as a signal of the stress experienced than as evidence that cadaverine is itself damaging.

Two nonexclusive interpretations follow. The first treats polyamine remodelling as a marker: a larger shift towards the diamines reflects greater catabolic activity and a more severe internal stress state. Cadaverine is of particular interest. This lysine-derived diamine is rarely measured in cereal heat studies, though it is known to increase under heat, drought, salt, and oxidative stress (Jancewicz et al., 2016; de Oliveira et al., 2020; Ozmen et al., 2023). In our panel, it separated the cultivars most clearly, which makes it a practical indicator. It may also act as a mediator: the same catabolism consumes the protective higher polyamines and generates hydrogen peroxide, thereby increasing the burden that the antioxidant and glyoxalase systems must counter. Consequently, catabolic ROS production coincides with declining enzyme activities in the sensitive cultivars, while restrained catabolism and sustained defences are mutually consistent in the tolerant ones (Napieraj et al., 2023; Samanta et al., 2023; Pál et al., 2015). The loss of about 58%–77% of spermine is especially consequential because spermine scavenges free radicals directly (Ha et al., 1998), protects plants from heat damage by inducing heat-shock-related genes (Sagor et al., 2013), and alleviates heat injury in wheat when applied exogenously (Jing et al., 2020); its depletion therefore removes a protective molecule when it is most needed (Napieraj et al., 2023). The direction of change is not universal: some wheat studies report moderate heat raising spermidine and spermine and lowering putrescine in a biosynthetic, acclimatory phase (Goyal and Asthir, 2010), whereas our 40 °C cultivars show the catabolic, damage-associated phase. The pool thus appears to follow a biphasic trajectory with stress dose, and a genotype’s position along it is itself an index of tolerance. At 40 °C, every cultivar had moved towards the catabolic, diamine-dominated end, and the size of that shift marks the most affected genotypes. This agrees with the ordination, in which the diamines align with the severe-heat pole and spermine with the control pole. The negative correlation between the diamines and markers of a healthy state (**Supplementary Figure S1**) reflects the temperature response shared by all cultivars; within a temperature, the diamines were unrelated to biomass, photosynthesis, or chlorophyll, although cadaverine tended to be lower in cultivars with higher glyoxalase I activity (**Supplementary Figure S2**). This relationship is an association rather than a demonstrated mechanism. The design profiles polyamine pools alongside oxidative and carbonyl markers in the same leaves but does not manipulate them, so a causal contribution cannot be separated from a correlated response. Establishing mediation would require exogenous higher polyamines, chemical inhibition of polyamine oxidase and diamine oxidase, which is known to perturb reactive oxygen and nitrogen homeostasis in salt-stressed tomato roots (Szepesi et al., 2023), or lines altered in polyamine catabolism, none of which was undertaken here. The marker role rests directly on the present measurements and is independent of whether mediation occurs.

The three tolerant cultivars reached this position by different strategies. Mv Lucilla shows the most balanced response, combining the highest chlorophyll retention with sustained antioxidant and carbonyl-detoxifying capacity, marked anthocyanin accumulation, and the lowest putrescine. The only exception is cadaverine, whose relative increase exceeds that of all other cultivars, although it starts from a low control value and reaches only an intermediate content. Mv Dandár relies most on carbonyl detoxification, retaining the highest glyoxalase I activity and one of the highest AOR/AER activities at 40 °C, with substantial proportional increases in APX and GR from a low baseline and low diamine levels. Mv Kolompos achieves tolerance by a distinctive, less enzyme-intensive route: superior carotenoid retention, uniquely stable DHAR and GST activities, and sustained glyoxalase I activity, which keeps its oxidative load low; its tolerance rests on these biochemical traits rather than on biomass retention, since its high control vigour gives it the largest proportional biomass loss of the panel. By contrast, Mv Tarsoly showed declines across all trait classes, while in Mv Ikva vigorous APX and GR induction is undermined by losses of GST, DHAR, and AKR; Mv Pirkadat is genuinely intermediate. The recurrence of this ranking across independent trait classes and in the multivariate and STI analyses (Hasan et al., 2024; Mohi-Ud-Din et al., 2021; Lamba et al., 2023) indicates that no single trait suffices and that coordination of pigment protection, antioxidant and carbonyl detoxification, and restrained polyamine catabolism separates tolerant from sensitive genotypes.

These results identify Mv Lucilla, Mv Kolompos, and Mv Dandár as candidate heat-tolerant germplasm for Central-European wheat breeding—a hypothesis requiring field validation—and point to a compact set of biochemical markers for screening (Zheng et al., 2025; Lamba et al., 2023). Some limitations should temper interpretation. The study was conducted at the vegetative stage under controlled chamber temperatures rather than under the shorter, more variable heat episodes experienced in the field, so the ranking should be validated against reproductive-stage performance, field performance, and grain yield (Balla et al., 2019, 2021). The heat treatments were applied under a 12/12 h light/dark cycle while the controls were grown under 16/8 h, a design chosen because heat episodes of this kind would occur in late autumn and early spring, when the daily light period is shorter. The 4-h reduction in light exposure under heat may itself have influenced photosynthesis and metabolism, and this should be taken into account when the responses reported here are interpreted. Direct measurements of oxidative and carbonyl-damage markers, together with measurements of the activities of the polyamine-catabolic enzymes PAO and DAO, would independently test the low-load tolerance strategy inferred here and confirm the catabolism-driven hydrogen peroxide production proposed above.

## 5 Conclusion

Heat tolerance in the six winter wheat cultivars did not depend on any single protective trait; rather, it was a dose-dependent outcome of how well each genotype coordinated its defences as temperature increased. Moderate heat mobilised the antioxidant, carbonyl-detoxification, and phenylpropanoid systems in every cultivar; at 40 °C, the genotypes differed in whether they sustained this mobilisation, indicating that the screening temperature determines what is observed (first objective). Mv Lucilla, Mv Kolompos, and Mv Dandár retained their pigments, largely sustained the activities of the ascorbate–glutathione and glyoxalase/AOR–AER enzymes, and showed only a moderate shift in the polyamine pool towards the diamines, whereas in Mv Tarsoly, these systems declined together. These biochemical traits—not gas exchange, which declined almost identically in all six cultivars—separated tolerant from sensitive genotypes (second objective). Polyamine remodelling indicated the severity of stress experienced and may add to it through the hydrogen peroxide released during catabolism. Two observations are new for winter wheat: cadaverine, a lysine-derived diamine seldom measured in cereal heat studies, separated the cultivars more sharply than any other single metabolite, and both arms of carbonyl detoxification decreased below control in one cultivar alone. A practical marker panel follows (third objective): sustained glyoxalase I and AOR/AER, maintained APX and GR, retained carotenoids, and low putrescine and cadaverine. The graded treatments also provide a basis for anticipating the effect of late-autumn and early-spring heatwaves on Central European winter wheat (fourth objective), provided that the shorter light phase applied under heat is taken into account. The next steps are to validate the panel across developmental stages and field environments, assess its relationship with grain yield, and dissect the regulation of polyamine catabolism. The study was conducted in growth chambers at one developmental stage without yield data; therefore, the three tolerant cultivars are candidate germplasm from a physiological screen, and no breeding recommendation can be made until the panel and ranking are confirmed in multiseason field trials.

## Data Availability

The datasets presented in this study can be found in online repositories. The names of the repository/repositories and accession number(s) can be found below: The raw data and the R scripts that reproduce all analyses and figures of this study are available at Zenodo (https://doi.org/10.5281/zenodo.22692577) and GitHub (https://github.com/yawarhabib/Genotype-specific-heat-responses-in-winter-wheat).

## Supplementary material

Supplementary Figure 1 Spearman rank correlation matrix among the measured growth, photosynthetic, pigment, antioxidant, carbonyl-detoxification, phenylpropanoid and polyamine traits. Ellipse shape and colour indicate the sign and magnitude of each pairwise correlation; values are shown for significant pairs. Coefficients are Spearman’s rank correlations across the 18 genotypes × temperature means.

Supplementary Figure 2 Spearman rank correlations among the traits within temperature. Each trait was centred on the mean of the six cultivars at each temperature before correlation, so that the coefficients describe covariation among cultivars independent of the temperature effect. Values are shown for pairs with p < 0.05 (t approximation with 14 degrees of freedom; 9 for pairs with anthocyanin).
