# Supplementary material for: Genotype-specific antioxidant, reactive-carbonyl, and polyamine responses underlying heat tolerance in six winter wheat cultivars
Source: Front Plant Sci. 2026 Oct 26;17:1973386. doi: 10.3389/fpls.2026.1973386 (PMC13746328; doi:10.3389/fpls.2026.1973386)
Supplement: Supplementary file 1 [file SupplementaryFile1.pdf]

Supplementary Material

Genotype-specific antioxidant, reactive-carbonyl, and polyamine responses underlying heat tolerance in six winter wheat cultivars

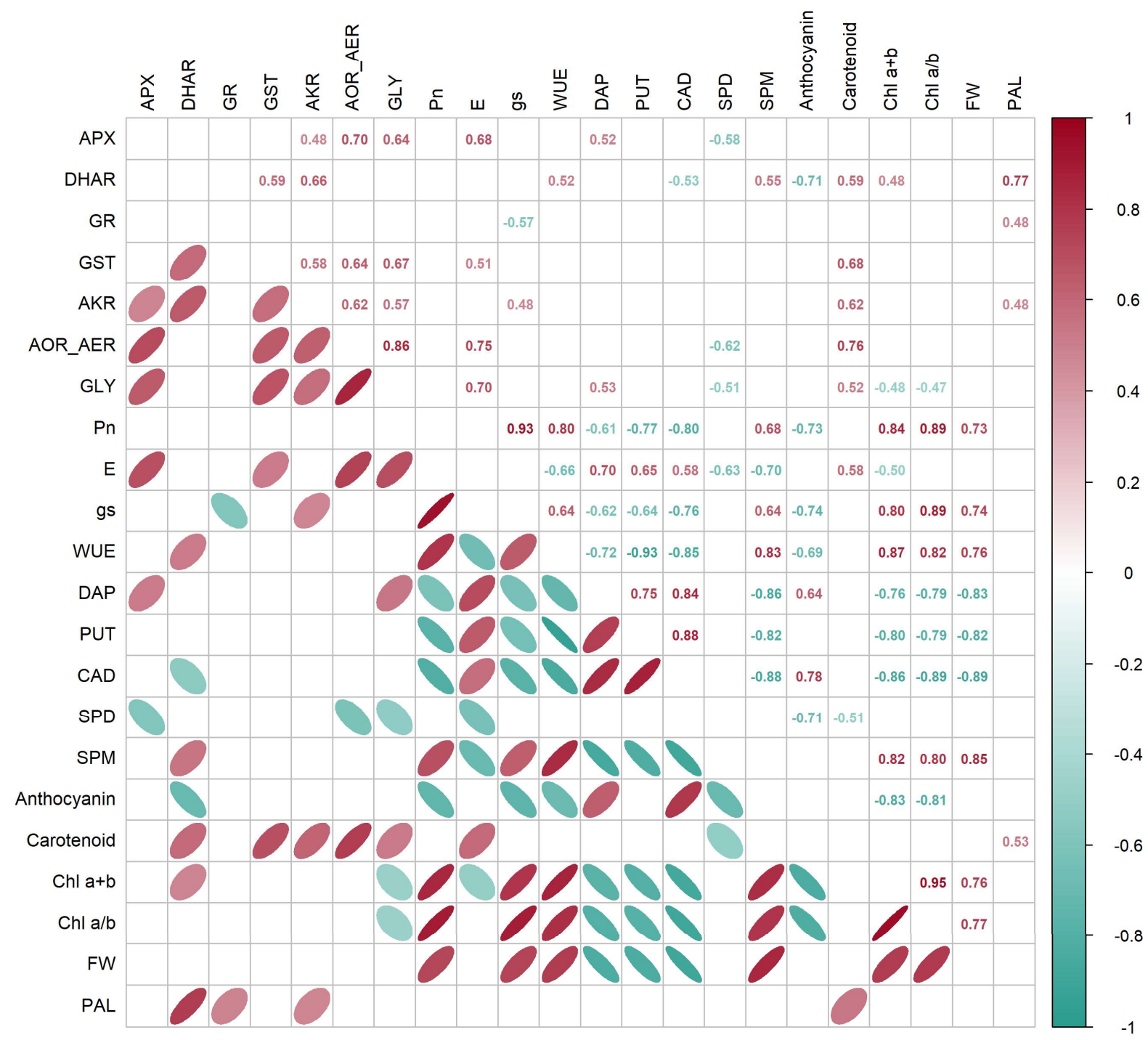

Supplementary Figure 1. Spearman rank correlation matrix among the measured growth, photosynthetic, pigment, antioxidant, carbonyl-detoxification, phenylpropanoid and polyamine traits. Ellipse shape and colour indicate the sign and magnitude of each pairwise correlation; values are shown for significant pairs. Coefficients are Spearman's rank correlations across the 18 genotype × temperature means.

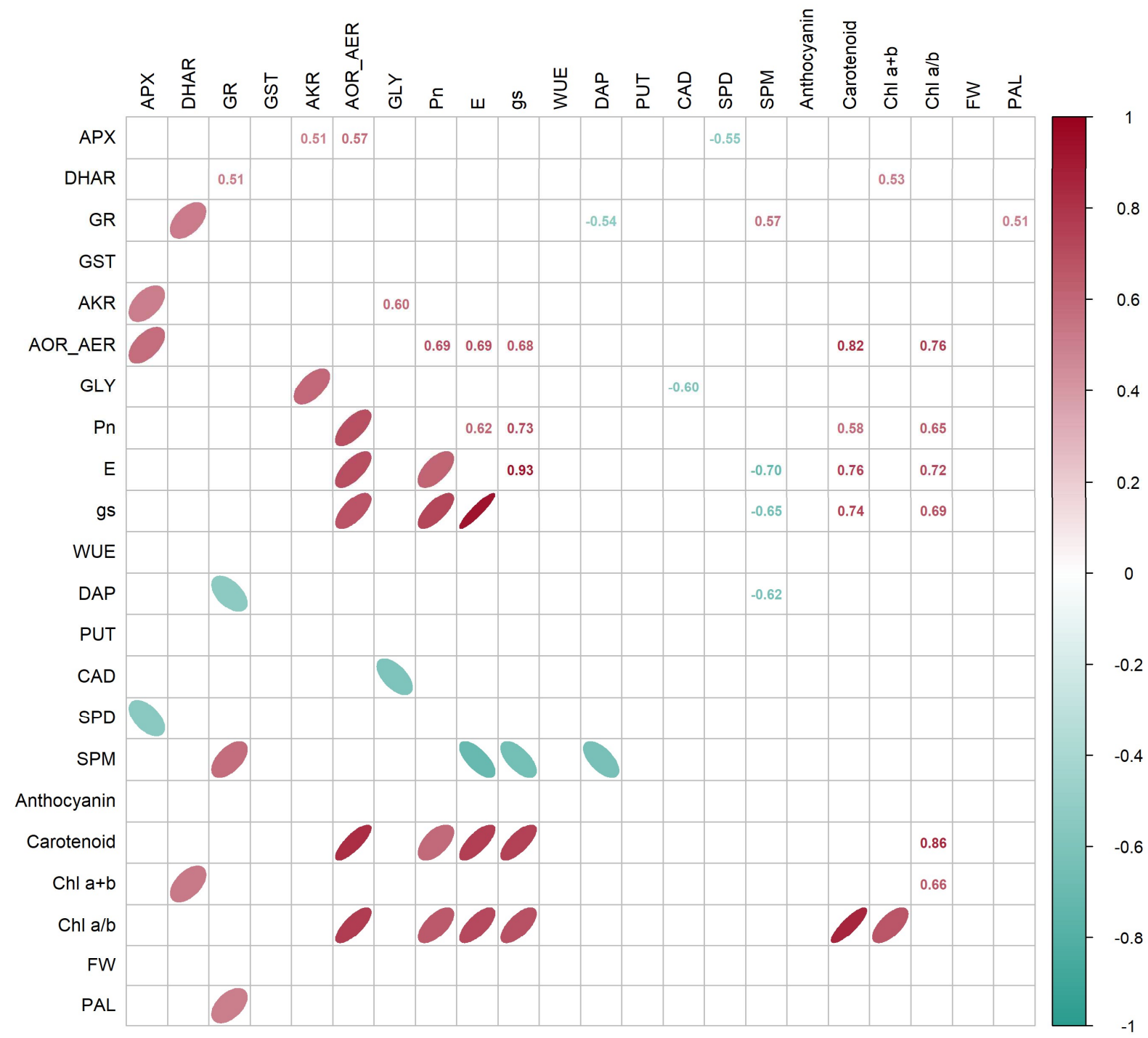

Supplementary Figure 2. Spearman rank correlations among the traits within temperature. Each trait was centred on the mean of the six cultivars at each temperature before correlation, so that the coefficients describe covariation among cultivars independent of the temperature effect. Values are shown for pairs with $p < 0.05$ (t approximation with 14 degrees of freedom; 9 for pairs with anthocyanin).
